# Zika Virus NS3 Protease Pharmacophore Anchor Model and Drug Discovery

**DOI:** 10.1038/s41598-020-65489-w

**Published:** 2020-06-02

**Authors:** Nikhil Pathak, Yi-Ping Kuo, Teng-Yuan Chang, Chin-Ting Huang, Hui-Chen Hung, John Tsu-An Hsu, Guann-Yi Yu, Jinn-Moon Yang

**Affiliations:** 1 Bioinformatics Program, Taiwan International Graduate Program, Institute of Information Science, Academia Sinica, Taipei, 11529 Taiwan; 2 Institute of Bioinformatics and Structural Biology, National Tsing Hua University, Hsinchu, 30013 Taiwan; 3 National Institute of Infectious Diseases and Vaccinology, National Health Research Institutes, Zhunan, 35053 Taiwan; 4 Institute of Biotechnology and Pharmaceutical Research, National Health Research Institutes, Zhunan, 35053 Taiwan; 5 Institute of Bioinformatics and Systems Biology, National Chiao Tung University, Hsinchu, 30010 Taiwan; 6 Department of Biological Science and Technology, National Chiao Tung University, Hsinchu, 30010 Taiwan

**Keywords:** Computational models, Virtual drug screening, Viral infection

## Abstract

Zika virus (ZIKV) of the *flaviviridae* family, is the cause of emerging infections characterized by fever, Guillain-Barré syndrome (GBS) in adults and microcephaly in newborns. There exists an urgent unmet clinical need for anti-ZIKV drugs for the treatment of infected individuals. In the current work, we aimed at the promising virus drug target, ZIKV NS3 protease and constructed a Pharmacophore Anchor (PA) model for the active site. The PA model reveals a total of 12 anchors (E, H, V) mapped across the active site subpockets. We further identified five of these anchors to be critical core anchors (CEH1, CH3, CH7, CV1, CV3) conserved across flaviviral proteases. The ZIKV protease PA model was then applied in anchor-enhanced virtual screening yielding 14 potential antiviral candidates, which were tested by *in vitro* assays. We discovered FDA drugs Asunaprevir and Simeprevir to have potent anti-ZIKV activities with EC_50_ values 4.7 µM and 0.4 µM, inhibiting the viral protease with IC_50_ values 6.0 µM and 2.6 µM respectively. Additionally, the PA model anchors aided in the exploration of inhibitor binding mechanisms. In conclusion, our PA model serves as a promising guide map for ZIKV protease targeted drug discovery and the identified ‘previr’ FDA drugs are promising for anti-ZIKV treatments.

## Introduction

The spread of Zika virus (ZIKV) from Asia and Africa to the Americas in recent years has raised alarms internationally. The ZIKV which was first identified in humans in 1952 in Africa, caused a 2015 epidemic outbreak in Brazil spreading to other countries becoming a serious health concern worldwide^1,2^. Thus in February 2016, ZIKV was declared a global public emergency by the WHO calling for urgent action against the viral infections^3^. ZIKV is a mosquito-borne virus from the family *Flaviviridae* alongside the Dengue virus (DENV), West Nile virus (WNV), Japanese encephalitis virus (JEV), Murray Valley encephalitis virus (MVEV), Yellow fever virus (YFV) etc.^4^. ZIKV infection could result in serious pathologies like induced fever, neurological implications like Guillain-Barré syndrome (GBS) in adults and neonatal microcephaly in newborns of infected pregnant women due to mother-to-fetus virus transmission^5^. The limited understanding of the ZIKV led to growing interest in the exploration of viral epidemiology, mechanisms of transmission-infection, clinical pathologies and prevention-treatment strategies by anti-viral vaccines and drugs^6^. However, the urgent need for treating infected patients, demands accelerated antiviral drug discovery which also needs to be robust against virus evolution.

The ZIKV genome consists of positive-sense RNA coding for three structural proteins (capsid C, prM/M and envelope E) forming virus components and seven non-structural proteins (NS1, NS2A, NS2B, NS3, NS4A, NS4B and NS5) functioning in various steps of the viral replication cycle^7^. Among ZIKV non-structural proteins, the NS2B/NS3 protease enzyme plays a key role in viral replication post genome-translation, by cleaving the single polyprotein precursor at specific sites to generate functional viral proteins. Thus the viral protease is considered an important and effective therapeutic target for preventing viral replication and infection^8–10^. The growing knowledge of ZIKV molecular biology was accompanied by increasing efforts in targeting the virus, with research works focusing on drug repurposing identifying various anti-ZIKV FDA drugs^11–13^ whose precise molecular targets are yet to be elucidated. Efforts focusing on ZIKV protease including the high throughput screening approaches have identified allosteric inhibitors^14–16^ with *in vivo* activities^16,17^ as well as few orthosteric inhibitor drugs^18,19^ with a molecule being active *in vivo*^19^. On the other hand, several structure-based approaches targeting ZIKV NS3 protease active site yielded inhibitors^20–22^ but it has proven to be challenging with only one drug molecule showing *in vivo* anti-ZIKV activity^23^ so far. Thus, a more comprehensive framework for targeting ZIKV NS3 protease active site is very much necessary to achieve effective viral protease inhibitor design and discovery with promise in clinical applications.

The current work employs a structure-based pharmacophore anchor approach that incorporates comprehensive interaction patterns of the target binding site, giving a robust hotspot model beneficial to explore target functional mechanisms and applicable in inhibitor discovery and optimization. This strategy proved to be fruitful in understanding protein-compound binding mechanisms previously^24–27^ and is applied to the ZIKV NS3 protease for studying consensus active site interactions and for inhibitor discovery via drug repurposing using FDA drugs. The ZIKV NS3 protease like some other flaviviral proteases has a flat, wide and charged active site posing a challenge for effective binding and competitive inhibition by small molecule inhibitors, thus needing novel targeting approaches^8^. Despite overall structural homology with other flaviviral proteases bearing a conserved chymotrypsin-fold, ZIKV protease contains, variable active site subpocket environments with negatively charged S1, S2 subpocket regions; unique substrate motifs like the ZIKV-specific substrate-binding regions at S3 subpocket^10,28^; salt bridges with NS2B cofactor residues absent in other flaviviral proteases^29^. We believe that for effective targeting of the ZIKV NS3 protease, knowledge of the protease active site anchor hotspots would be highly beneficial. Thus we created a ZIKV protease Pharmacophore Anchor (PA) model with consensus interactions of active site residues with interacting compound moeities represented as ‘anchors’ with features like anchor interaction types, anchor residues and anchor moiety preferences. The PA model was then employed for *in silico* ‘anchor-enhanced virtual screening’, a step-wise approach for screen inhibitors using anchors, progressing from our previous work on DENV protease where an anchor-based scoring function was used^27^.

## Results

### Overview of the workflow

First and foremost, we pursued a sequence-structure analysis examining our target ZIKV NS3 protease. Sequence analysis involved multiple sequence alignment (MSA) of the ZIKV NS3 protease and NS2B cofactor domains (African strain MR766) with corresponding sequences from other mosquito-borne flaviviruses like DENV, WNV, JEV and MVEV followed by building phylogenetic trees (refer to Materials and methods: Multiple sequence alignment) summarized in Supplementary Fig. S1A. A significant global alignment of ZIKV NS2B cofactor and NS3 protease chains with the homologous counterparts is seen, most of the aligned residuesbeing highly conserved (residues colored in blue) with many conserved sequence motifs with other viral proteases, however, it still contains some unique residue patterns. For example, in the NS2B MSA, we find ZIKV protease conserved TGxS, RLDV, LDxxG and unique VEED motifs (underlined orange in Supplementary Fig. S1A). In the NS3 MSA, ZIKV protease shows highly conserved motifs shared with other flaviviruses like GVYR, GTSGSPI (with catalytic Ser135) and GLYGNGV; motifs with highly conserved residues like QxGVGVM, FHTxWHxTxGA (with catalytic His51), LxPYWGxVKxD (with catalytic Asp75); and motifs like LLAVPPGERAR with specific residues. The NS2B cofactor and NS3 protease MSA-based phylogenetic trees also show ZIKV protease being evolutionary close but not in the same branch with DENV, WNV, JEV and MVEV proteases, highlighting its overall conservation and retained specificities. Such similarities/differences are also reflected in ZIKV vector preferences for *Aedes* mosquitoes like DENV, in its neurological pathologies alike to that of WNV, JEV, and MVEV meanwhile ZIKV uniquely induces microcephaly in the new-borns of infected pregnant women. The growing interest in the ZIKV NS3 protease have resulted in its crystallization in various forms^30–35^, summarized in the Supplementary Fig. S1B. All the crystal structures belong to two distinct ZIKV protease conformations, an open and a closed form as previously reported^10^. In the open forms, the flexible NS2B cofactor C-terminal region does not extend into the active site, leading to an incomplete cavity with missing subpockets (as in 5T1V, 5TFN and 5TFO of Supplementary Fig. S1B). In the closed forms, the NS2B cofactor folds into a β-hairpin completing the active site (as in 5GJ4 and 5LC0) facilitating substrate/inhibitor binding. As the closed form being ideal for studying binding mechanisms and inhibitor discovery, for the current work we chose 5GJ4 containing a bound peptide ligand TGKR at the protease active site^31^.

**Figure 1:**
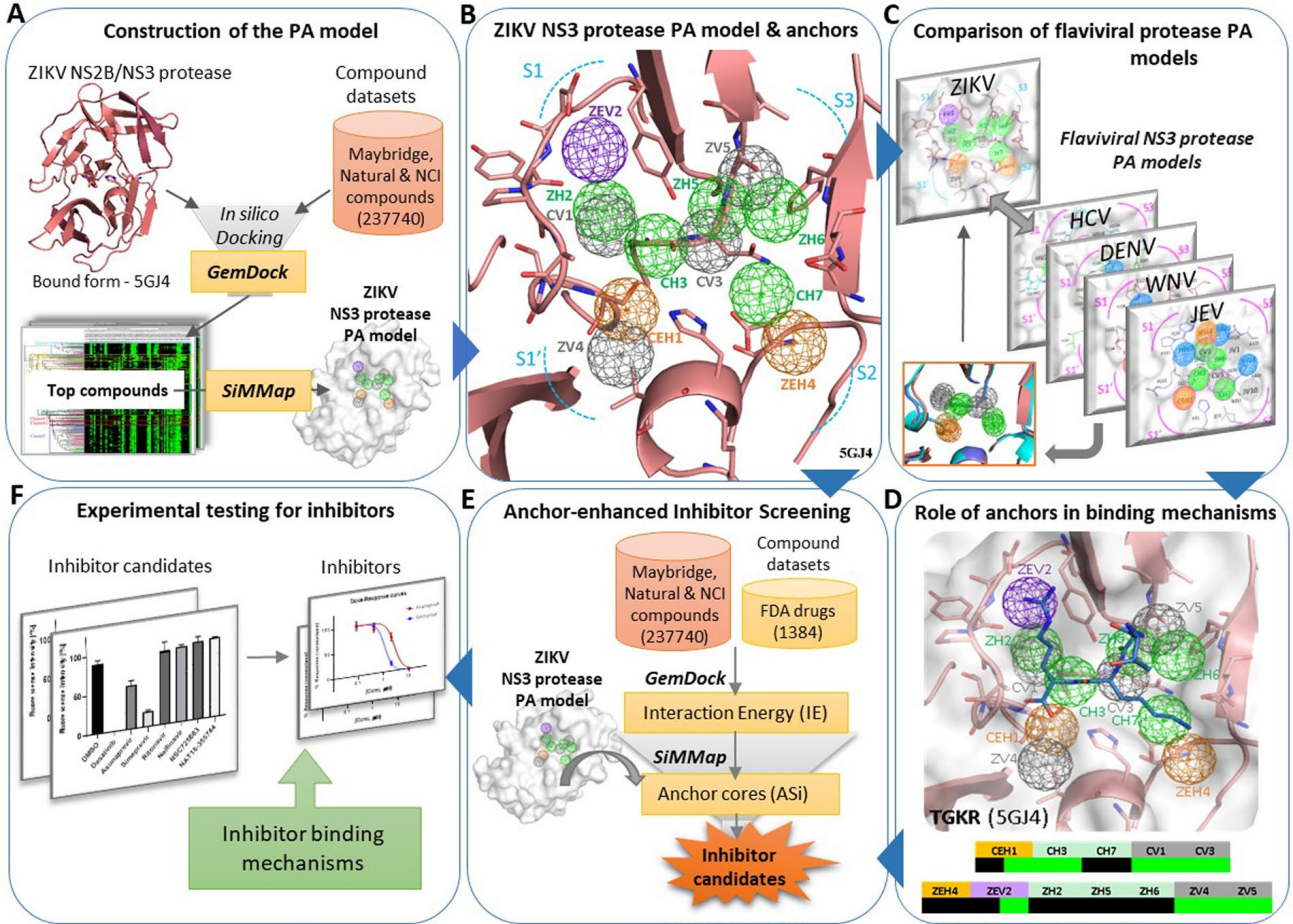
Overview of the workflow.

Figure 1 summarizes the overview of the current workflow on the construction of the ZIKV NS3 Protease PA model and its application in inhibitor discovery. For constructing the PA model, we first docked large compound datasets into the protease active site using GemDock^36^ (Fig. 1A, also see Materials and methods: Protein-compound dataset preparation and docking). The interactions of the top compounds were summarized using SiMMap^37^ as pharmacophore anchors, resulting in a 3D Pharmacophore anchor (PA) model of the ZIKV NS3 protease. We then explored the PA model and its Electrostatic E, Hydrogen bonding H, van der Waals V anchors located across the protease sub-pockets (Fig. 1B). The ZIKV protease PA model was compared with those of other flaviviral proteases (Fig. 1C) and matching anchors were evaluated. As shown in Fig. 1D, the role of ZIKV protease anchors in inhibitor/substrate binding mechanisms were also explored. In the next step, the PA model anchors were applied to inhibitor discovery through anchor-enhanced virtual screening to yield ZIKV inhibitor candidates (Fig. 1E). Finally, experimental testing by *in vitro* assays yielded active inhibitors, whose mechanisms were further explored (Fig. 1F).

### ZIKV NS3 protease Pharmacophore Anchor (PA) model

**Figure 2:**
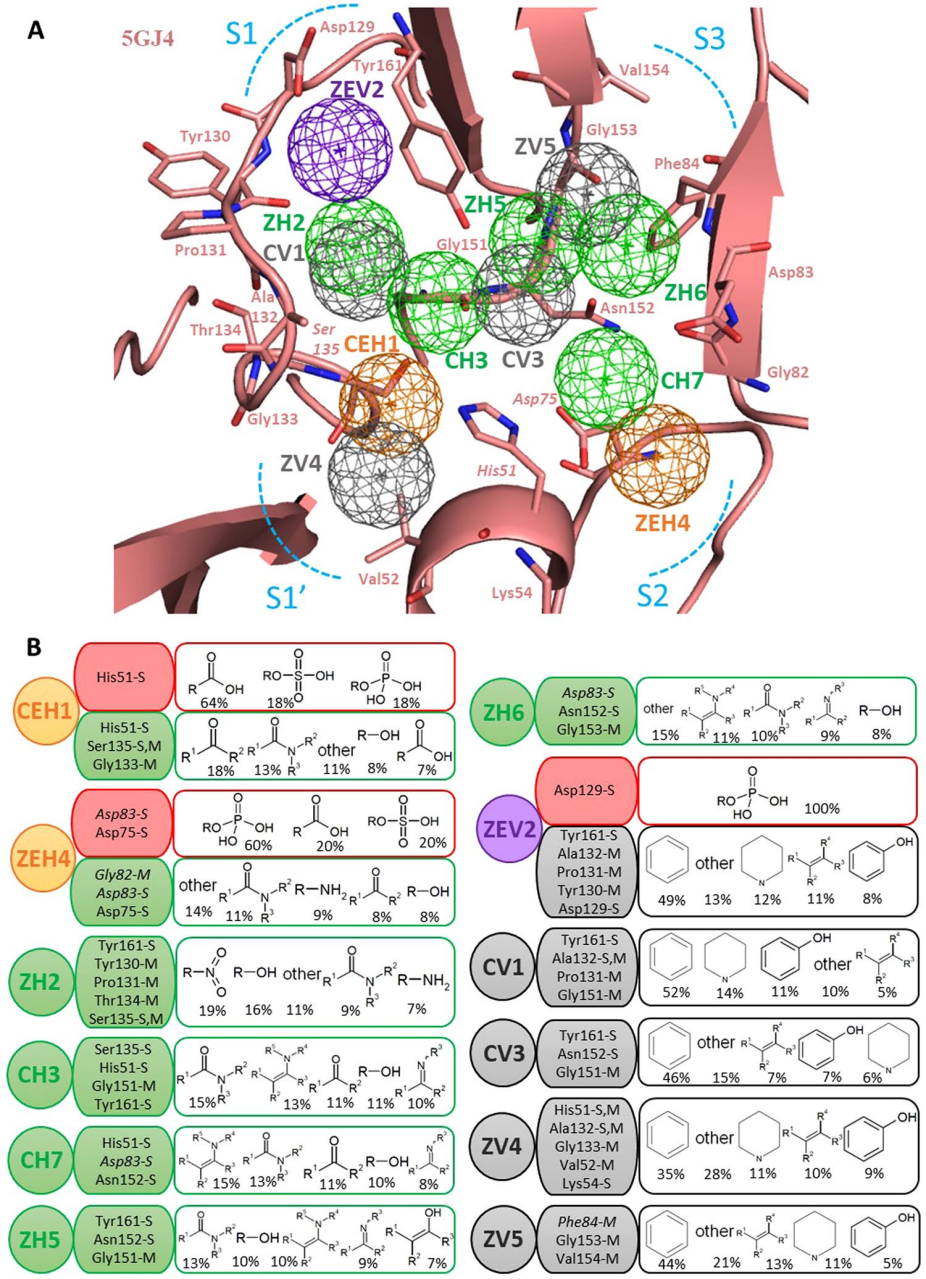
The ZIKV NS3 protease Pharmacophore Anchor (PA) model - anchors and their features. (**A**) The ZIKV NS3 protease active site (5GJ4) with anchors (mesh spheres) at subpockets S1′, S1, S2, S3 (blue dotted curves) with anchor residues (sticks). The anchors are colored as E-red, H-green, V-grey, EV-purple, HV-orange. (**B**) The anchor features, interaction-types E-H-V or mixed, anchor residues with interacting side chain S & main chain M atoms (NS2B cofactor residues in italics), anchor moiety preferences showing top five prefered functional groups (with % among all interacting moieties).

A PA model consists of a protein binding site with embedded anchors, representing consensus interactions between the protease residues and the functional groups of interacting compounds. The anchors feature an interaction type (E-electrostatic, H-hydrogen bonding, V-van der Waals) which sometimes merge to form mixed anchors supporting multiple interaction types. Other anchor features include supporting anchor residues (involved in interactions) and anchor moiety preferences (preferred compound functional groups at the anchor). The PA model of ZIKV NS3 protease active site (from 5GJ4) shown in Fig. 2A consists of twelve distinct pharmacophore anchors distributed across the protease sub-pockets S1′, S1, S2, and S3, including five Hydrogen bonding anchors (H), five van der Waals anchors (V) and three mixed anchors (EH or EV). The mixed anchor CEH1 is obtained by merging of anchors E1-H1; ZEH4 by merging of E3-H4 and ZEV2 by merging of E2-V2 anchors respectively, following the ‘anchor-matching rules’ (refer to Materials and methods: Building the PA model and calculating anchor scores). The anchor features of the PA model anchors: anchor types, anchor residues and five most preferred moieties are summarized in detail in Fig. 2B.

We observe a mixed anchor CEH1 adjacent to the S1′ subpocket at the oxyanion hole, supporting both E and H interactions (Fig. 2A). The E-interactions involve the catalytic anchor residue His51 side chain (positively charged) engaging with negatively charged compound moieties, the majority being R-COO^−^, R-SO4^−2^, and R-PO4^−2^ groups constituting 64%, 18% and 18% of all interacting groups respectively (Fig. 2B). Here the His51 side chain, Ser135 side & main chain and Gly133 backbone atoms involve in H-bonding with ketones at 18% and amide groups at 13% being the top two preferred moieties. Among the H-bonding anchors, CH3 anchor is formed by catalytic Ser135, His51 & non-catalytic Gly151, Tyr161 residues that prefer amide, enamine, carboxyl amide, hydroxyl and imine moieties. The ZH2 anchor in deeper S1 subpocket supports H–bonding interactions of Tyr161, Tyr130, Pro131, Thr134 and catalytic Ser135 with nitro, hydroxyl, carboxyl amide and amine functional groups accounting to 19%, 16%, 9% and 7% of all groups respectively (Fig. 2A,B). The S3 subpocket supports anchors ZH5 (with Tyr161, Asn152 and Gly151) and ZH6 (with Asp83, Asn152 and Gly153) both having moiety preferences for carboxyl amide, hydroxyl, imine and enamines. At subpocket S2, anchor residues His51 & Asn152 of NS3 chain and Asp83 of NS2B co-factor form CH7 anchor which preferes enamines (15%) and carboxyl amides (13%). We observe two prominent mixed anchors ZEV2 and ZEH4 occuring deep inside S1 and S2 subpockets respectively. The ZEV2 anchor supports dual interactions, negatively charged Asp129 forming E-interactions with compound R-H_2_PO_4_ groups and H-bonding of Tyr130, Pro131, Ala132 and Tyr161 residues with aromatic ring moieties. The ZEH4 anchor (with NS3 Asp75, NS2B Asp83 and Gly82) is a dense negatively charged anchor hotspot formed by the two aspartic acids interacting with favorable compound moieties (Fig. 2A,B). This anchor additional offers H-bonding inetractions with amide, amine, ketone and alcohol functional groups. These electrostatic anchors agree with the negatively charged landscape of ZIKV protease S1 & S2 subpockets accommodating P1 & P2 charged groups as previously established^10^. Amongst the hydrophobic anchors, we notice ZV4 anchor at the S1′ subpocket supported by catalytic His51 and neighboring Ala132, Gly133, Val52 and Lys54 residues interacting with favoring hydrophobic aromatic and heterocyclic rings (Fig. 2A,B). Another V-anchor CV1 (with Tyr161, Ala132, Pro131 and Gly161) binds with aromatic, heterocyclic, phenolic and other hydrophobic groups. We also observe a CV3 anchor (with Tyr161, Gly151, and Asn152) and a ZV5 anchor (with Phe84, Gly153, and Val154) at subpocket S3 both of them preferring same top five groups: aromatic rings, heterocyclic rings, alkenes, phenols and other hydrophobic moieties. Thus our PA model gives a complete interaction hotspot map of ZIKV protease active site revealing anchors as sites of key binding interactions.

### ZIKV NS3 protease PA model compared to other flavivirus proteases

The exploration of ZIKV is recent, compared to other flaviviruses like the DENV and WNV that have been studied for well over a decade now. Thus it would be wiser to apply our knowledge and learning from flaviviruses like DENV, WNV, JEV and HCV, etc., to better understand and target the current issue of ZIKV. Thus we progressed from the preliminary flaviviral protease sequence-structure analysis discussed previously, to directly compare and contrast the virus protease PA models. First, the current ZIKV protease PA model was overlapped with previously built DENV, WNV, JEV and HCV protease PA models^27^ by aligning NS3 protease active sites using structure-based CE alignment^38^. Then anchors amongst proteases were matched following ‘anchor-matching rules’ (explained in Materials and methods: Building the PA model and calculating anchor scores) as depicted in Supplementary Fig. S2. We identified five ZIKV protease anchors that matched across all proteases CEH1, CH3, CH7, CV1, CV3 (so the prefix ‘C’, magenta dotted circles & arrows in Supplementary Fig. S2A,B) to be the flaviviral core anchors, a conserved trademark of the flaviviral NS3 proteases^27^. The remaining anchors ZEH4, ZEV2, ZH2, ZH5, ZH6, ZV4, ZV5 not conserved across all proteases are viewed as ZIKV specific anchors (so the prefix ‘Z’, brown dotted arrows in Supplementary Fig. S2B). We futher closely examined the ZIKV protease core and specific anchors with anchors from closely related DENV and WNV proteases (Supplementary Fig. S3).

The five core anchors are perfectly overlapped among the viral protease PA models with matching anchor features (see Supplementary Fig. S3A). For instance, the oxyanion hole core anchor CEH1 in ZIKV with anchor residues His51, Ser135 aligns perfectly with the corresponding CEH1 from DENV and WNV with same top five preferred moieties for E & H interactions; the Gly133 anchor residue in ZIKV and DENV corresponding to Thr132 in WNV still retaining the anchor. The CH3 anchor matches among three proteases with aligned anchor residues and shared anchor preferences, depicting strong conservation of the core anchor. The CH7 anchor among ZIKV, DENV and WNV PA models shares anchor residues His51 and Asn152; while in ZIKV the NS2B Asp83 substituted for Asp75 of DENV and WNV compensating the H-bonding interactions. The core anchor CV1 is conserved with residue 131 being Pro in ZIKV & WNV and Lys in DENV; residue at 132 being Ala in ZIKV, Pro in DENV & Thr in WNV (Supplementary Fig. S3A), all anchor residues being part of conserved sequence motif GTSGSPI described before in sequence analysis. Another core anchor CV3 upheld by Gly151 in ZIKV and Asn153 in DENV and WNV; with additional van der Waals interactions by His51 and Gly153 with matching preferred moieties (Supplementary Fig. S3A). The interesting observation is that variable core anchor residues do not change the anchor hotspot environments that must be critical in function and robust against resistance mutations.

We proceeded to evaluate the specific anchors of ZIKV, DENV and WNV proteases (Supplementary Fig. S3B). Among the ZIKV specific anchors some reveal ZIKV-specific binding environments, while others reveal matching interactions with that of DENV and WNV. The ZEV2 anchor aligns with DHV4-WHV8, with key Asp129 anchor residue in ZIKV enabling electrostatic interactions at the negatively charged S1 subpocket while DHV4 and WHV8 anchors support hydrogen bonding. However, these anchors offer van der Waals interactions (Tyr161, Pro131, Pro/Ala132 and others) with four preferred groups in all three viral proteases. At S2 subpocket, the ZEH4 aligns with DH9 and WHV4 anchors offering common H-bonding interactions through conserved catalytic Asp75, Asp83 of ZIKV or Asn84 of WNV (but not Thr83 of DENV). Moreover, ZEH4 E-interaction by charged anchor residue Asp83 is unique to ZIKV (not in others) agreeing with some previous observations on ZIKV protease Asp83^29^. We find another set of matching ZH2-DH2-WH5 H-anchors with aligning Ser135, Thr134 and Tyr130/Phe130 residues (in Supplementary Fig. S3B). Near subpocket S3, matching ZH5-DH5-WH2 anchors highlight the conserved subpocket binding environments. We also observe overlapped hydrophobic ZV5-DV8-WV6 anchors supported by Phe84 in ZV5, Phe85 in WV6 and Met84 (and Thr83) in DV8. In close vicinity an anchor ZH6 aligns to WH6 with matched Asn152, Gly153 from NS3 and Asp83/Asn84 from NS2B cofactor. At the S1′ subpocket adjacent to the oxyanion hole, there are aligned ZV4 and DV6 anchors with His51 and Val52 residues. At S1′ subpocket in DENV protease, a DE2 anchor by Arg54 is uniquely observed without a corresponding ZIKV anchor due to a lesser positive Lys54. In summary, comparative analysis of core and specific anchors is crucial to learn about precise similarities/differences in interactions across ZIKV-DENV-WNV proteases and their role in substrate recognition, proteolysis and inhibitor binding mechanisms, valuable in the pan-virus/virus-specific inhibitor discovery.

### Evaluation of the protease PA model by evolutionary conservation and binding mechanisms

Our ZIKV protease PA model anchors representing active site interactions would be critical during functional and binding mechanisms, thus must have evolutionary significance. Hence we proposed to explore anchors in relation to evolution, through anchor residue conservation profiles. We grouped ZIKV NS3 protease residues into four sets: core anchor residues - all core anchor residues (including catalytic triad), specific anchor residues - all specific anchor residues but not in core anchors, binding site residues - all non-anchor residues in the binding site cavity and other residues - all remaining protease residues. We used the ConSurf server to obtain residue evolutionary conservation scores^39^ for the four sets and analyzed % of residues with each residue conservation score for core and specific anchor residues, binding site residues and other residues. In Supplementary Fig. S4A, we see that more than 50% of anchor residues (combined core and specific) show the highest conservation score of 9, thus reflecting higher importance of anchor residues most likely due to their anchor interactions. On further evaluation of core and specific anchor residues conservation (Supplementary Fig. S4B), we notice more than 50% of both core and specific anchor residues having the highest conservation score of 9, while the remaining anchor residues showed good conservation scores of 6–8. A slightly higher percentage of core anchor residues than specific anchor residues have a score of 9 which could be due to core anchor preservation across flaviviruses. The lower conservation scores of some anchor residues is due to the involvement of residue main chain atoms in anchor interactions that are conserved in spite of residue mutation during evolution. All these findings show the evolutionary conservation of anchor residues validating our PA model.

**Figure 3:**
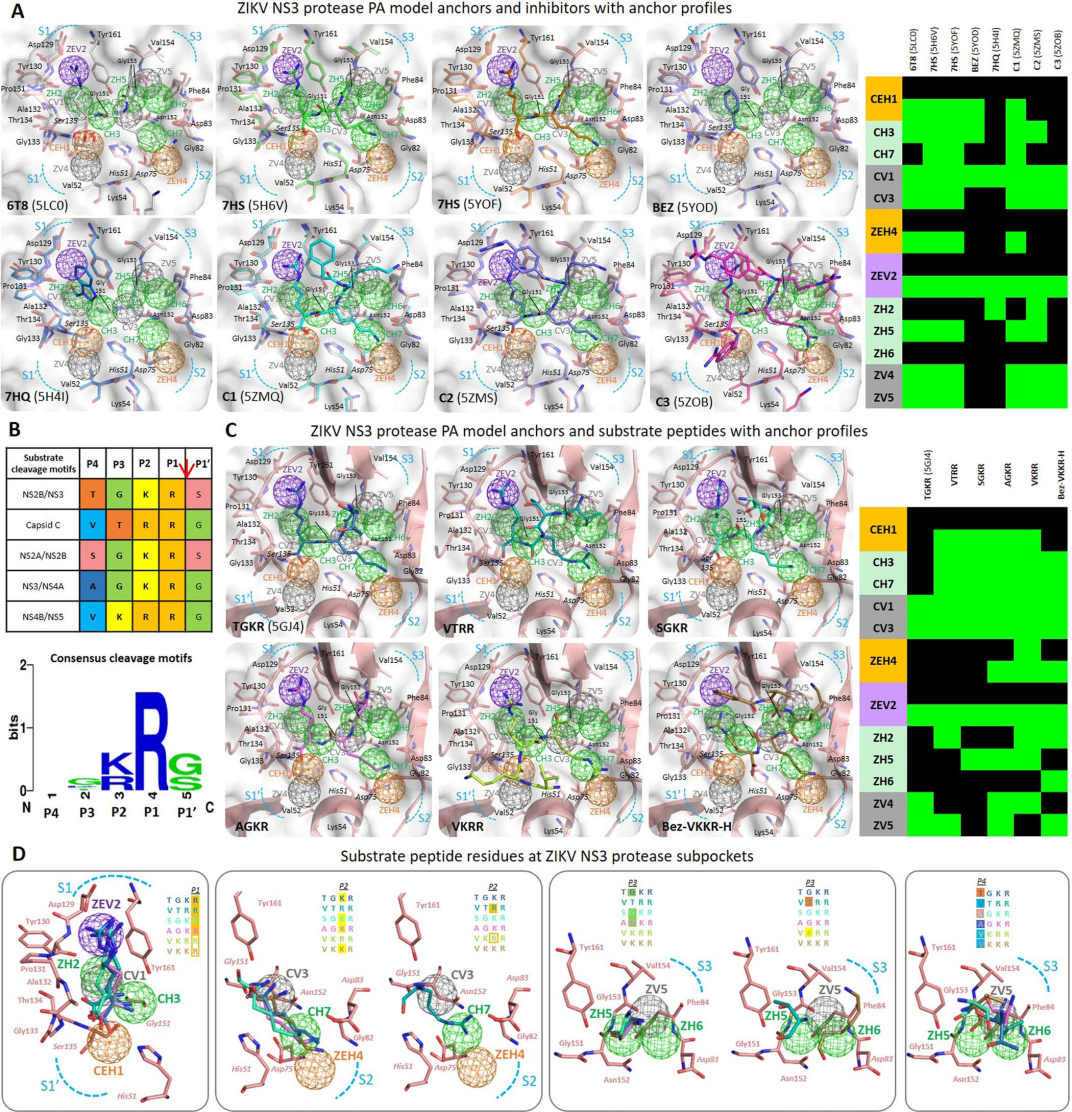
ZIKV NS3 protease PA model anchors with inhibitors and substrate peptides. (**A**) The crystal structures of eight inhibitor-bound ZIKV NS3 proteases overlapped with the protease PA model depicting active site subpockets (surface), anchors (mesh spheres), residues and inhibitors (both shown in sticks). The anchor occupancy profiles of inhibitors are shown as heat-map (green – anchor interaction, black - no interaction). (**B**) Summary of ZIKV NS3 protease substrate cleavage motifs in the genome polyprotein showing P4-P3-P2-P1↓P1′ residues; Web logo showing consensus residues at cleavage motifs. (**C**) ZIKV NS3 protease PA model with P4-P1 substrate peptides, crystal structure for bound peptide TGKR (5GJ4) and docked poses for substrate peptides VTRR, SGKR, AGKR, VKRR and Bez-VKKR-H are displayed; the anchor profiles of the substrate peptides are displayed as heat-map. (**D**) At each subpocket, the binding substrate peptide residues P1, P2, P3 and P4 are seen occupying the anchors.

We proceeded to examine the significance of the viral protease active site anchors and their involvement in binding mechanisms of inhibitors and substrate peptides, whose functional groups interact with protease residues (inolved in anchors). The PA model was applied to the ZIKV NS3 protease crystal structures from PDB: 5LC0 (6T8), 5YOF (7HS), 5YOD (BEZ), 5H4I (7HQ), 5H6V (7HS), 5ZMQ (C1), 5ZOB (C2) and 5ZMS (C3) which were aligned to our 5GJ4 ZIKV protease PA model (Fig. 3A). Inhibitor anchor occupancy scores and profiles were determined (refer to Materials and methods: Building the PA model and calculating anchor scores) and the inhibitor chemical moieties occupying the anchors via interactions with anchor residues were summarized (in Supplementary Table S1A). Consider the boronic inhibitor 6T8, a covalently bound substrate mimetic showing a sub-micromolar inhibition efficacy^20^, whose functional groups are occupying nine PA model anchors (anchor profile in Fig. 3A). Near subpocket S1′, the 6T8 cyclic diester (boronic acid-glycerol) engages the CEH1 anchor by fulfilling the anchor’s preferences for acidic groups and the ZV4 anchor via its cyclic ring (Fig. 3A). At the S1 subpocket, the 6T8 Arg-like group fulfills CV1 and ZEV2 anchors via charged interactions of Asp129 with its positive guanidinium group. The 6T8 -CO-NH- group is binding to CH3 anchor (preferring amide moiety) and the aminomethyl-phenyl-acetyl group is engaging CV3 anchor (and also ZEH4). The strong agreement of 6T8 crystal pose with our PA model indicates anchors’ role in inhibitor binding. Another dipeptide inhibitor 7HS in crystal poses 5H6V and 5YOF with proteases from two virus lineages, occupies all five core anchors and specific anchors ZEH4, ZEV2, ZH5, ZV4 and ZV5 (details in Supplementary Table S1A). At CEH1, the 7HS carboxyl functional group binds strongly to catalytic Ser135 in both the poses, the only difference in 5YOF is an interaction of Ala132 main chain -C=O instead of Gly133 -NH- in 5H6V & 5GJ4 retaining the anchor and overall binding pose (Fig. 3A). This suggests that anchors are unaffected by change or displacement of residue atoms being more robust and dependable than individual residues. In 5YOD, inhibitor BEZ engages CEH1, CH3, CV1 and ZEV2 anchors by benzoyl moiety showing van der Waals interactions with Tyr161, Ala132 of CV1. Another molecule 7HQ (known as EN300) in 5H4I binds deeply in the S1 subpocket with CV1 and ZEV2 residues Tyr161, Ala132 and Pro131 by hydrophobic/π-π stacking interactions, while inhibitor -OH moiety fits ZH2 (Fig. 3A, Supplementary Table S1A). The more recent structures 5ZMQ, 5ZMS, and 5ZOB contain bound peptidomimetic inhibitors C1 (phenylacetyl-Lys-Lys-Arg-COOH), C2 (4-guanidinomethyl-phenylacetyl-Lys-Lys-Arg-H) and C3 (4-guanidinomethyl-phenylacetyl-Arg-Arg-Arg-4-amidinobenzylamide) with P1′-P1-P2-P3-P4 groups binding to corresponding protease sub-pockets^40^. For C1 and C2, at CEH1 anchor the –COOH group is electrostatically interacting with His51 and H-bonding to Gly133, while for C3 a P1′ benzylamide group is occupying CEH1 and ZV4 anchor extending into the S1′ subpocket (Fig. 3A, Supplementary Table S1A). At ZEV2, Asp129 anchor residue locks-in Arg groups of C1, C2 and C3 via E-interactions. The P2 Lys groups of C1 & C2 and P2 Arg group of C3 engage with Asp83 of NS2B cofactor occupying S2 subpocket ZEH4 anchor (Fig. 3A) while the P4 groups of C1, C2 and C3 extend out without any anchor occupancies. These observations verify our PA model anchors and their involvement in inhibitor binding mechanisms.

The ZIKV protease functions by proteolytic site-specific cleavage of genome polyprotein which involves recognition and binding of substrate peptides at the active site sub-pockets. The ZIKV NS3 protease recognizes P4-P3-P2-P1-P1′ substrate peptides connecting functional viral proteins^41^ summarized in the table and consensus logo in Fig. 3B. We notice a strictly conserved P1 Arg, a positively charged P2 Arg or Lys and other varying peptide residues. To deeply explore underlying binding mechanisms of the substrate peptides VTRR, SGKR, TGKR, AGKR and VKRR, our PA model was employed. Firstly, the substrate peptides were built, binding poses were obtained from docking (Materials and methods: Protein-compound dataset preparation and docking) except for TGKR (5GJ4) with crystallized binding pose; binding features like interaction energies, anchor occupancies were determined (Materials and methods: Building the PA model and calculating anchor scores) and results summarized in Fig. 3C,D and Supplementary Table S1B. In the crystal pose 5GJ4, a P4-P3-P2-P1 substrate peptide Thr-Gly-Lys-Arg (TGKR) is binding to the protease subpockets S4-S3-S2-S1 respectively. The P1 Arg –COOH is engaging core CEH1 anchor bonding to residues Ser135, Gly133 and His51; the P1 Arg group fits S1 subpocket with alkyl chain at CV1 and positively charged guanidinium group bonds to negatively charged Asp129 at ZEV2 anchor. This P1 Arg binding event involving anchors (CEH1, CV1 & ZEV2) is consistently observed across the substrate peptides (Fig. 3D) and is critical to orient P1-P1′ substrate -CO–NH- bond at oxyanion hole for catalytic cleavage^31^. At the S2 subpocket, binding of TGKR P2 Lys is through alkyl chain interaction with hydrophobic CV3 and bonding of terminal amine at CH7 and ZEH4 anchors which is also observed in SGKR and AGKR substrate binding. For other substrates like VTRR the P2 Arg binding at S2 is via anchors CV3, CH7 and strong anchoring at ZEH4 is due to additional E-interactions by Asp75 of NS3 and Asp83 of NS2B locking the charged Arg group (Fig. 3D). Thus, we identify the recognition and binding of multiple substrate residues are enabled through anchors with preferences for multiple moiety types. Also, at S3 subpocket we find P3 residue backbone atoms of Gly (in TGKR, SGKR, AGKR) and Thr (in VTRR) H-bonding with ZH5 anchor residues Tyr161 –OH and Gly153 –NH- atoms (Fig. 3C,D). The variable residues at substrate P4 like Thr, Val, Ser, Ala etc., do not occupy any anchors as they extend out from protease active site, thus allowing for residue non-specificity (Fig. 3D). Furthermore building on the ZIKV protease substrate profiling work that identified amino acids V, K, K and R as the most preferred P4, P3, P2 and P1 substrate amino acids^42^, we analyzed binding mechanisms of Bez-V-K-K-R (a Bez-P4-P3-P2-P1 ligand bound to WNV NS3 protease in PDB: 2FP7) with ZIKV NS3 protease with anchors (Fig. 3C). Its overall binding pose at subpockets is similar to reference TGKR like P1 Arg at S1 and P2 Lys at S2, however, P1 Arg side chain interacts with ZH2 and is not buried deeply at ZEV2 anchor which is hydrophobically gripped by the backward extending ligand Bez ring. The P3 Lys engagement at S3 subpocket via anchors ZH6 and ZV5 (not seen in VKRR) and the P4 Val binding via ZV5 anchor (like in VTRR) are also observed here. In summary, all the findings show that ZIKV NS3 protease recognizes and binds to varying substrate peptides by subpocket anchors which offer flexible interactions with diverse moiety types, further verifying the PA model anchors and their importance in target binding.

### Application of the protease PA model for ZIKV drug discovery

**Figure 4:**
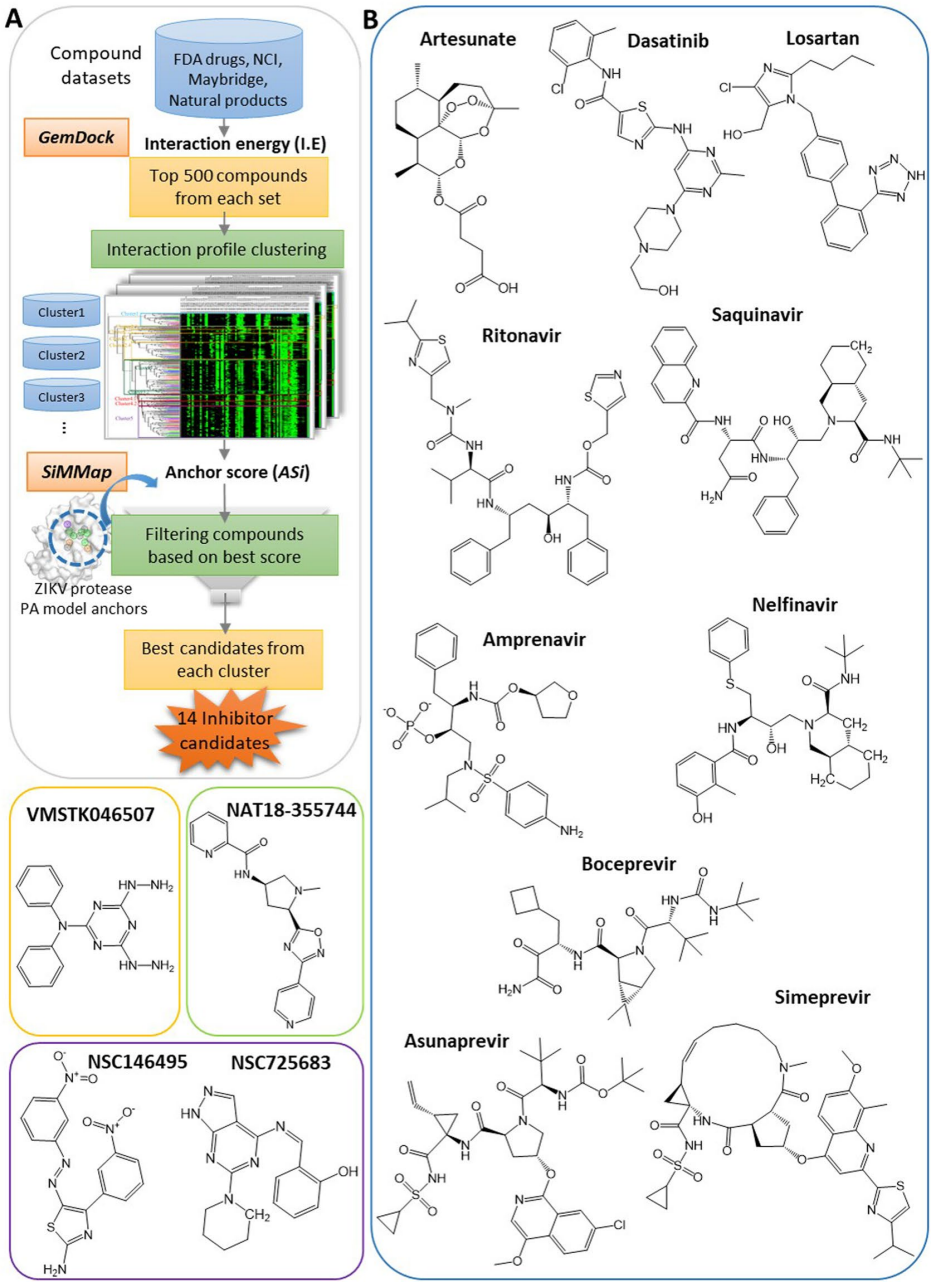
Anchor-enhanced virtual screening and inhibitor candidates. (**A**) Stepwise virtual screening using ZIKV PA model anchors to obtain inhibitor candidates. (**B**) 2D structures of 14 inhibitor candidates including 10 FDA drugs (blue outline), one Maybridge compound (orange outline), one natural product (green outline) and two NCI compounds (purple outline).

Employing pharmacophore anchors of protein drug targets for discovering inhibitors has been established as a viable approach in previous studies^25–27^. Currently, we targeted ZIKV protease by deploying its PA model anchors in drug discovery, through a stepwise *in silico* virtual screening approach called ‘anchor-enhanced virtual screening’ (flowchart in Fig. 4A). Unlike regular docking energy-based virtual screening, anchor-enhanced virtual screening additionally considers anchor scores, thus prioritizing compounds with higher anchor occupancies as effective inhibitors. In this approach, FDA drug set, NCI compounds, Maybridge compounds and Natural products were docked into protease active site and the poses were analyzed to select top 500 compounds with best interaction energies (I.E) from each set first. Their compound-residue interaction profiles were clustered based on similarity (in atom composition and interaction patterns) to obtain distinct compound clusters. Next, anchor scores (AS_i_) of the compound clusters were determined (details in Materials and methods: Building the PA model and calculating anchor scores) followed by score-based filtering of representative compounds with best anchor scores from each cluster as inhibitor candidates. A total of 14 inhibitor candidates were obtained including several FDA drugs, two NCI compounds, one Maybridge compound and a Natural product (shown in Fig. 4B). Their binding poses and interactions with ZIKV protease PA model anchors are shown in Supplementary Fig. S5, while detailed binding features like interaction energies, anchor scores and compound moieties at respective anchors are summarized in Supplementary Table S2. We observe that these inhibitor candidates bind to ZIKV protease active site at distinct subpockets engaging multiple anchors at with anchor scores ranging from 7 to 10 showing good inhibitory potential.

### Analysis of inhibitor antiviral activities by *in vitro* assays

**Figure 5:**
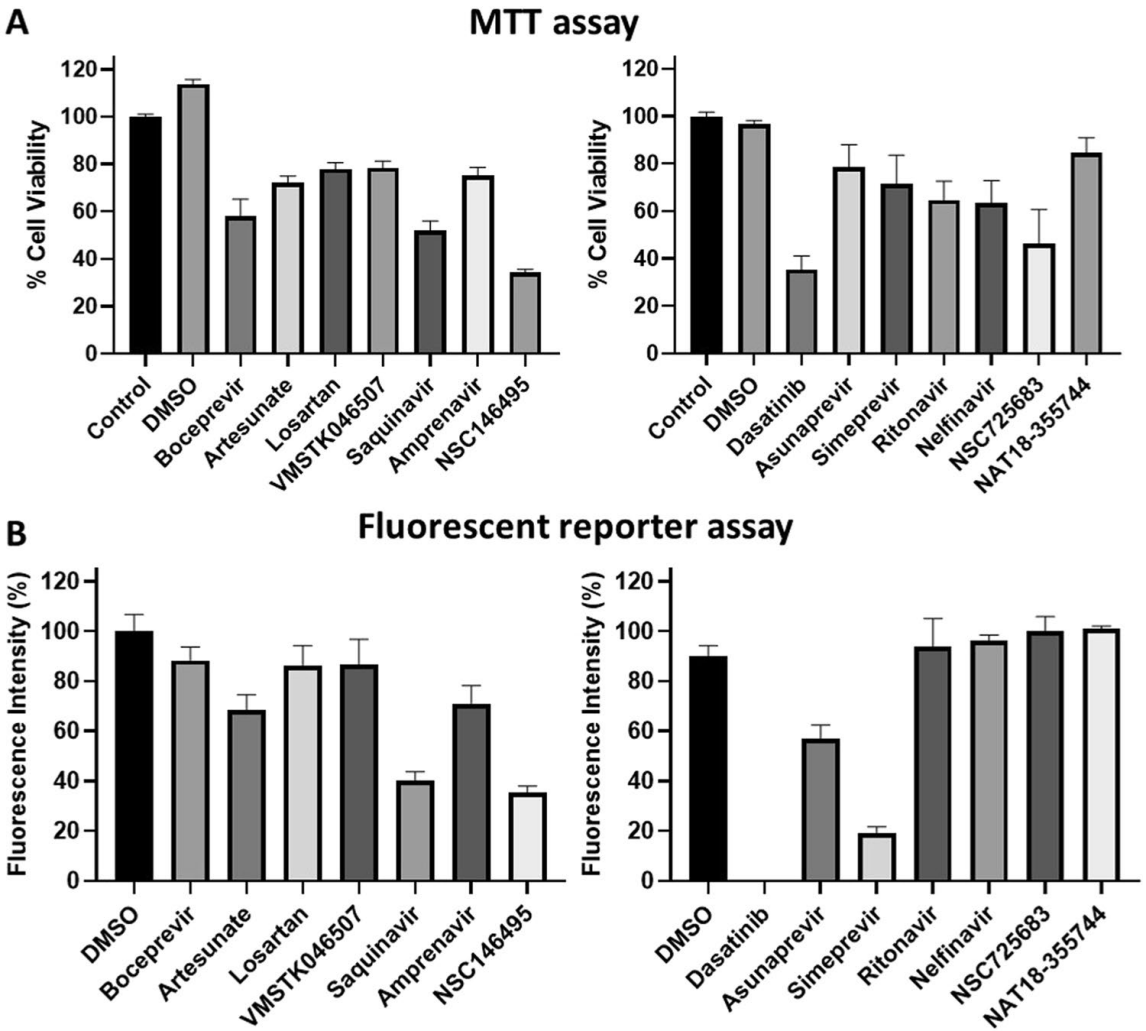
Analysis of anti-ZIKV activities of inhibitor candidates. (**A**) MTT assay for evaluation of the cytotoxicities of the inhibitor candidates at 5 µM concentration, post-treatment and incubation for 24 hours. (**B**) Candidate compounds tested for activity against ZIKV by Fluorescence reporter assay, where the infected cells were incubated with 5 µM of compounds for 24 hours. The fluorescence intensities measured correspond to the amount of ZIKV.

Experimental assays serve as a key means for establishing effectiveness of potential antivirals. We primarily tested the antiviral activities of inhibitor candidates using a fluorescent reporter assay which were further quantified by a virus production assay, both of which analyzed inhibitory effects on *in vitro* ZIKV replication. First of all, we evaluated the 14 inhibitor candidates for cytotoxicities by MTT assay, where the primate Vero76 cells were treated with 5 µM of inhibitor candidate compounds (and DMSO) and % cell viabilities were measured (details in Materials and methods: MTT assay), the results summarized in Fig. 5A. Then, the ZIKV fluorescent reporter assay was employed for testing comopund antiviral activities, where viral replication was quantified by the intensity of a fluorescent protein-tagged virus (MR766-Venus) for treatment vs control (details in Materials and methods: ZIKV fluorescent reporter assay). The tested candidates (at 5 µM concentration) that retained the cell viability greater than 60% in MTT assay (compared to control) are considered to be non-cytotoxic (Fig. 5A) and those that decreased the fluorescence intensities less than 60% (compared to DMSO) are considered to have anti-ZIKV activities (Fig. 5B). As an effective antiviral inhibitor must be non-cytotoxic along with antiviral activities; according to the current results Saquinavir, NSC14649, Dasatinib, Asunaprevir, and Simeprevir showed anti-ZIKV activities (shown by decreased fluorescence intensities) amongst which only Asunaprevir and Simeprevir were non-cytotoxic. Thus Asunaprevir and Simeprevir showing promise as non-toxic effective ZIKV inhibitors were further evaluated.

**Figure 6:**
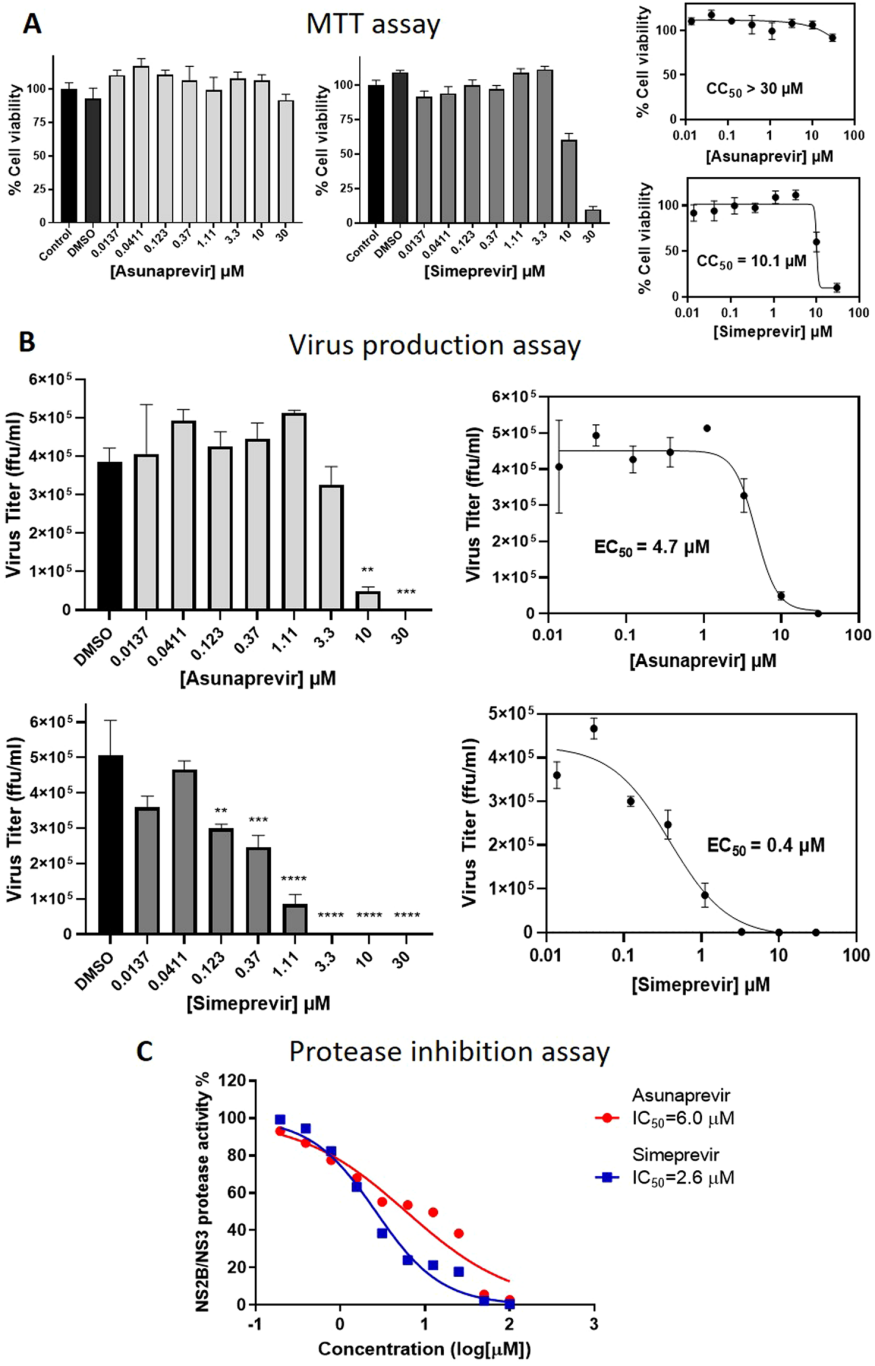
Quantification of inhibitor activities. (**A**) In MTT assay for cytotoxicities, Asunaprevir and Simeprevir were tested at various concentrations by measuring the cell viabilities (by Absorbance at O.D., 570 nM) after inhibitor treatment and 24-hour incubation; further curves were plotted to obtain CC_50_ values. (**B**) ZIKV production assay measuring inhibitor antiviral activities, in which U-87 MG cells were infected with ZIKV (PRVABC-59, MOI = 1), treated with various inhibitor doses and incubated for 24 hours. The residual virus in the titer was quantified by measuring the viral focus forming units (ffu/ml); the statistical significance versus DMSO (**p < 0.0021, ***p < 0.0002, ****p < 0.0001) by Dunnett’s multiple comparison test was represented. The results plotted as dose-inhibition curves and inhibitor EC_50_ values were calculated. (**C**) ZIKV NS2B/NS3 protease inhibition assay to assess the protease activity inhibition by Asunaprevir and Simeprevir at multiple doses. The residual protease activities in the presence of inhibitors were measured, sigmoidal curves were plotted and the IC_50_ values were calculated.

To confirm and quantify the inhibitor antiviral activities, we plan to perform dose-dependent inhibitor testing for Asunaprevir and Simeprevir at eight different concentrations. The inhibitor cytotoxicities at these doses (and DMSO) were checked by MTT assay using human U-87 MG cells (see Materials and methods: MTT assay) where % cell-viabilities were plotted as dose-cell viability curves from which cytotoxic concentration 50% (CC_50_) values were calculated (Fig. 6A). Asunaprevir with CC_50_ > 30 μM is observed to be non-cytotoxic at all the tested doses and Simeprevir with CC_50_ value of 10.1 μM, is non-cytotoxic below 10 μM. We then employed the ZIKV production assay, in which virus-infected U-87 MG cells were treated with multiple doses of Asunaprevir and Simeprevir and incubated (details in Materials and methods: ZIKV production assay). The residual virus was then quantified by measuring the virus titer as virus focus forming units (ffu/ml) in the supernatant for inhibitor treatments compared to DMSO, which were then plotted as dose-inhibition curves for determining the inhibitor efficacies EC_50_ values (Fig. 6B). From the results, we observe a significant reduction in the virus titer for Asunaprevir treatment from a dose of 3.3 µM and up to 30 µM; while for Simeprevir treatment a significant decrease in virus production was noticed at 0.123 µM and higher doses, all compared to DMSO. For Simeprevir, the reduction in viral titer at higher doses 10 µM & 30 µM must be carefully considered, as the decrease in viral count could also be partially due to a decrease in the viable cells at these concentrations. The results as a whole, show an increased virus inhibition with inhibitor dose, suggesting their dose-dependent anti-ZIKV activities. Thus we plotted sigmoidal dose-inhibition curves and determined inhibitor antiviral efficacies with EC_50_ values, 4.7 µM for Asunaprevir and 0.4 µM for Simeprevir respectively (Fig. 6B). Thus, the *in vitro* testing of 14 screened inhibitor candidates discovered and established Asunaprevir and Simeprevir FDA drugs as anti-ZIKV inhibitors with dose-dependent antiviral activities and EC_50_ values in the lower micromolar range.

The antiviral activities of the two inhibitors were established by cell-based assays, however, it is important to further establish that the inhibitors are indeed targeting the viral protease as predicted by the PA model. For this, we pursued an *in vitro* FRET-based ZIKV NS2B/NS3 protease inhibition assay which measures the changes in % protease activity based on the fluorescence from the proteolytic cleavage of a quenched substrate benzoyl–Nle–KKR–AMC by purified NS3 protease in the presence of inhibitors, inspired from published protocols^43,44^ (more details in Materials and methods: ZIKV NS2B/NS3 protease enzyme expression, purification and enzyme inhibition assay). The current inhibitors Asunaprevir and Simeprevir were tested at ten different concentrations for the inhibition of ZIKV protease activity, the results were plotted as dose-inhibition curves in Fig. 6C. The results show that for Asunaprevir and Simeprevir as compound concentration increases % protease activity decreases showing dose-dependent protease inhibition. The inhibitory potencies represented as IC_50_ values were determined for Asunaprevir and Simeprevir to be 6.0 µM and 2.6 µM respectively. Thus Asunaprevir and Simeprevir can be established as effective anti-ZIKV protease inhibitors with micromolar inhibitory potencies.

### Binding mechanisms of discovered inhibitors

**Figure 7:**
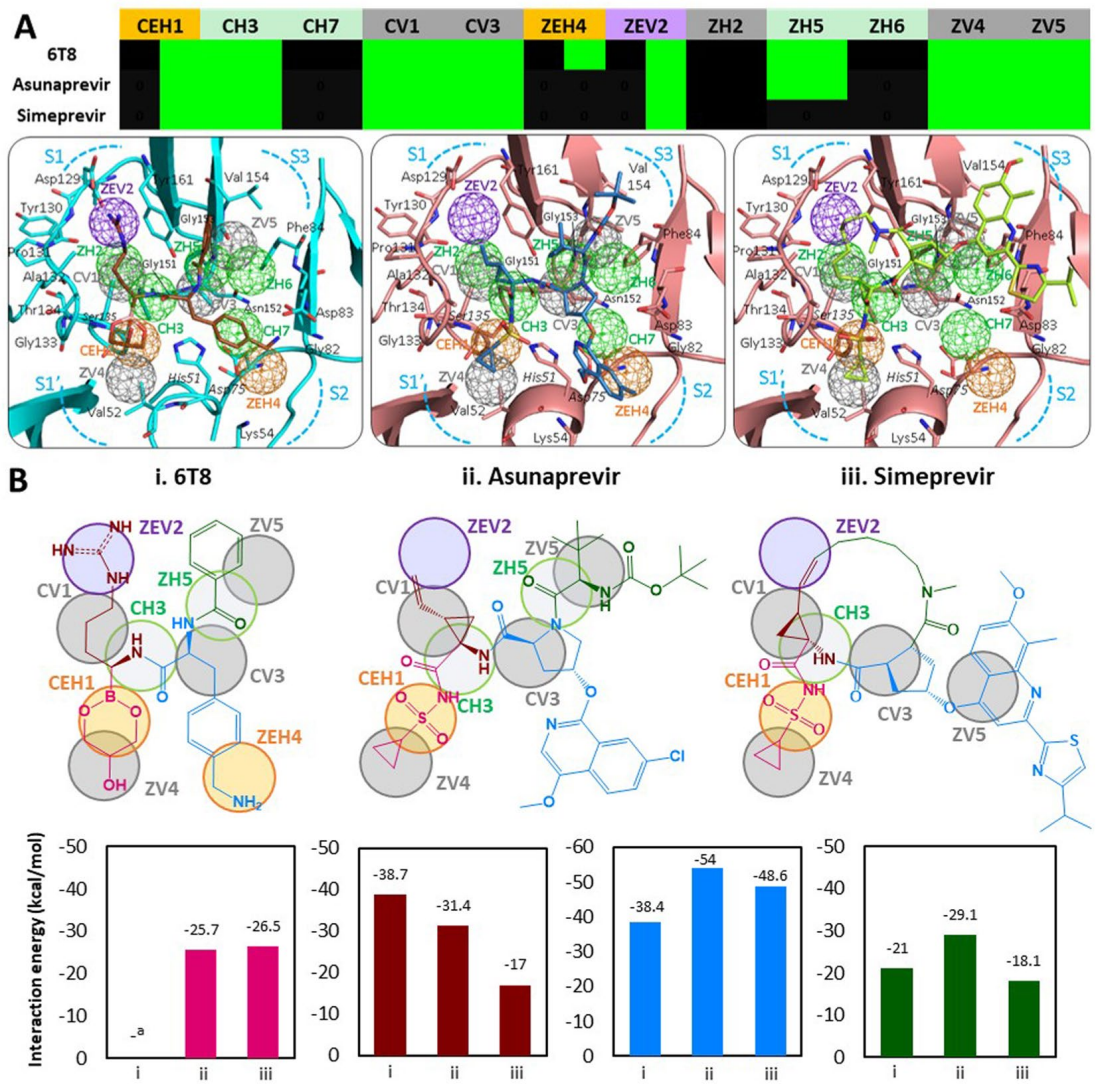
Analysis of inhibitor binding mechanisms for Asunaprevir and Simeprevir. (**A**) Anchor occupancy profiles for Asunaprevir, Simeprevir and known inhibitor 6T8 (green – interaction, black - no interaction). Inhibitor binding models with ZIKV NS3 protease for Asunaprevir, Simeprevir and 6T8 (in 5LC0). (**B**) 2D structures of, i. Asunaprevir, ii. Simeprevir and iii. 6T8 showing inhibitor substructures (colored magenta, brown, blue and green) occupying the anchors. Comparison of interaction energies by corresponding substructures from three inhibitors. ^a^interaction energy scoring function not applicable (due to covalent bonding of 6T8 moiety to Ser135).

After observing anchor involvement in binding mechanisms of crystallized inhibitors and substrate peptides, we decided to investigate the binding mechanisms of currently discovered ZIKV protease inhibitors, Asunaprevir and Simeprevir through anchors. Here, we also included a known crystallized inhibitor 6T8 (from PDB 5LC0) as a reference for comparison. The binding of Asunaprevir, Simeprevir (docking poses in 5GJ4) and 6T8 (crystal pose in 5LC0) with ZIKV protease depict inhibitor moieties engaging anchors by interacting with anchor residues at the protease subpockets, also summarized in anchor profiles (heatmap in Fig. 7A). On closer examination of inhibitor binding poses and anchor occupancy profiles, we notice similar underlying inhibitor anchor relationships. All the inhibitors Asunaprevir, Simeprevir and 6T8 occupied core anchors CEH1, CH3, CV1 & CV3 and specific anchors ZEV2, ZV4 & ZV5 of the PA model; both Asunaprevir and 6T8 occupy an additional ZH5 anchor, while only 6T8 engages an extra ZEH4 anchor (anchor profiles in Fig. 7A). This is because Asunaprevir is binding to S1′, S1, S2, and S3 subpockets compared to Simeprevir occupying S1′, S1 and S3 but not extending into the S2 subpocket; while the known inhibitor 6T8 is occupying all the four subpockets (binding poses in Fig. 7A). The specific inhibitor moieties interacting at each anchor are detailed in Supplementary Table S1A for 6T8 and in Supplementary Table S2 for Asunaprevir and Simeprevir. In general, for large-sized inhibitors containing numerous functional groups, exploring precise functional group contributions to resulting binding/activity is a challenge. We addressed this by analyzing inhibitor substructures (a cluster of chemical moieties) for their involvement in anchor occupancies and contributed interaction energies (in Fig. 7B). The inhibitor 2D structures with their occupied anchors are depicted, showing four corresponding substructures (based on atom composition and binding patterns, differentially colored) binding to S1′-S1-S2-S3 protease subpockets and the inhibitor substructure interaction energy contributions summarized as bar graphs in Fig. 7B.

Consider inhibitor magenta substructures which are engaging CEH1 anchor of S1′ subpocket, by the sulfonyl groups in Asunaprevir & Simeprevir and by corresponding boronic acid group of 6T8 which uniquely forms a covalent bond with Ser135 at the anchor (Fig. 7A,B). Also, the 6T8 glycerol group (forms a cyclic diester ring) and the cyclopropane rings of Asunaprevir and Simeprevir satisfy the nearby hydrophobic ZV4 anchor. We also analyzed the magenta substructure interaction energies (calculated by GemDock function) in Asunaprevir binding pose to be −25.7 kcal/mol and in Simeprevir binding pose to be −26.5 kcal/mol, almost equal due to same fragments interacting at same anchors. It must be noted that the energy function used here could not be applied to 6T8 and its magenta substructure due to its covalent bonding, unaccountable in the calculation (bar graphs in Fig. 7B). The brown inhibitor substructures are interacting with the S1 subpocket anchor CH3, CV1 and ZEV2. For all three brown substructures, the amide –NH- group anchors to CH3, while the hydrophobic alkyl chains bind to CV1 anchor. Among substructures engaging the mixed ZEV2 anchor, the 6T8 terminal 4-guanidino group is buried deeply into S1 by hydrophobic and charged interactions, analogous hydrocarbon chains of Asunaprevir and Simeprevir only involve in ZEV2 hydrophobic interactions as they lack a charged functional group. Among blue substructures binding at subpocket S2, 6T8 has a 4-(aminomethyl)phenyl group (occupying CV3 & ZEH4 anchors), while Asunaprevir has a pyrrolidin-1-yl moiety at CV3 anchor and a (7-chloro-4-methoxyisoquinolin-1-yl)oxy group extending into the S2 subpocket. The Simeprevir blue substructure consists of a cyclic 5-membered ring (occupying CV3) corresponding to the Asunaprevir moiety, but with a 4-(propan-2-yl)-1,3-thiazol-2-yl]quinolin-4-yl}oxy group is uniquely extending into the S3 subpocket engaging ZV5 anchor. The blue substructures of Asunaprevir and Simeprevir with multiple aromatic rings are bulkier than that of 6T8, thus having higher interaction energy contributions of −54 kcal/mol and −48.6 kcal/mol respectively (Fig. 7B). The green substructures of 6T8 and Asunaprevir occupy specific ZH5 and ZV5 anchors at S3 subpocket, unlike the cyclic groups of Simeprevir. Moreover, Asunaprevir green substructure has an additional amide ester functional group compared to 6T8 reaching out into protease S3 subpocket contributing to the interaction energy of −29.1 kcal/mol. The total interaction energies of inhibitor binding poses of Asunaprevir is −135.8 kcal/mol and Simeprevir is −110.3 kcal/mol. Summarizing the observations, we infer that Asunaprevir with an anchor score of 8 and Simeprevir with a score of 7 are closeby to reference inhibitor 6T8 having an anchor score 9; all inhibitors occupying the core anchors CEH1, CH3, CV1, and CV3 and some specific anchors. The magenta substructures near S1′ contain negative charged acidic groups (E-interacting type) in previrs and 6T8 that engage flaviviral protease core CEH1 anchor by bonding to anchor residue catalytic Ser135, believed to be indispensable for the protease inhibitory activity^27^. Moreover, the inhibitor interactions at CEH1 are further stabilized by engaging of ZV4 anchor by the substructure hydrophobic groups (cyclopropane in –previrs, glycerol in 6T8) (Fig. 7A). From the above inhibitor substructure analysis, we observe that the anchor-directed binding mechanisms of Asunaprevir and Simeprevir are similar to that of reference 6T8 inhibitor, establishing them as promising ZIKV NS3 protease inhibitors. Several crucial insights from the inhibitor binding mechanisms in this study, like the critical determinants of viral protease inhibition will step up the anti-ZIKV drug discovery.

## Discussion

The relatively recent emergence of ZIKV as a global epidemic sparked interest in the infectious disease mechanisms and targeting of the viral components like the current ZIKV protease could lead to effective antivirals^9^. However, there are gaps in extensive knowledge of the viral protease, in terms of structural conformations (linked or unlinked forms), substrate recognition mechanisms and viable inhibitors as of yet^29^. For example, quality data on the importance of protease residues in functional and binding interactions like residue mutation studies can help us identify critical active site residues for targeting, but such information is currently unavailable. To boost the ongoing research focusing on the drug target ZIKV NS3 protease, a systematic and comprehensive analysis of the viral protease binding site landscape to understand functional mechanisms and to discover potent inhibitors was done, leading to successful anti-ZIKV drugs. Our current work, the ZIKV NS3 protease PA model is a crucial for comprehensive understanding of the molecular binding mechanisms of the protease via anchors. The PA model reveals critical anchor residues, their role in binding site interactions (with substrates or inhibitors) and their targeting by inhibitor/compound functional groups, making our work a key stepping stone for targeting ZIKV protease through inhibitor discovery.

The PA model is a pharmacophore anchor map representing the consensus summary of the active site interactions obtained after the docking of 243,943 compounds (from multiple datasets) covering diverse chemical space to capture the chemical groups preferred in anchor interactions. However in some instances, like for the negatively charged E-anchor ZEV2 in our model phosphate group is the only prefered moiety found, mostly due to lack of other positively charged moieties in analysed interacting compounds. The anchors depicting conserved interactions of protease residues must be functionally important, cross-checked by evaluation of residue conservation which revealed higher evolutionary preference of anchor residues. To explore the role of anchors in active site functional and inhibition mechanisms, we proceeded to analyze binding mechanisms of ZIKV protease inhibitors (co-crystallized) and substrate peptides through anchor interactions, residues and moiety preferences. Surprisingly many of the 12 PA model anchors were endorsed by the limited number of available crystallized inhibitors and analyzed substrate peptides. Importantly these anchors, unoccupied and unverified by the current inhibitors/substrates, point out to novel anchor-guided modifications for improved inhibitor affinities by lead optimization. For instance, the ZH2 anchor deep in the S1 subpocket is rarely engaged and can be effectively targeted with preferred compound moieties via H-bonding. Harnessing some novel anchors like the ZH6 anchor, enable binding with NS2B cofactor residues at S3 subpocket improving affinity for the design of effective protease inhibitors. The importance of anchors across protein families was examined by comparison of the PA model of ZIKV protease with that of DENV and WNV proteases; identifying the critically conserved flaviviral core anchors (a trademark of the flaviviral proteases^27^) and ZIKV specific anchors. The PA model anchors applied to anchor-enhanced virtual screening yielded inhibitor candidates of which two previr drugs successfully showed anti-ZIKV activities by protease inhibition *in vitro*, thus projecting anchors as a promising tool in drug discovery. Moreover, similarities in binding/inhibition mechanisms of Asunaprevir and Simeprevir with reference inhibitor 6T8 were revealed through anchor and inhibitor substructures. For some inhibitors crystallized with ZIKV protease, like the 6T8 which employed covalent bonds in their binding interactions technical limitations were encountered while elucidating protease binding mechanisms. The GemDock energy function that calculated interaction energies of compound poses in the protein cavity, is based on bonding parameters for E-H-V interaction types^45^ and does not take covalent bond into account due to lacking parameters, thus failing to calculate interaction energies of 6T8 and its substructures forming covalent bonds. Another technical issue is for binding mechanisms of substrate peptides, that contain repeating peptide backbone and sidechain groups tend to bind to multiple similar anchors (like CH3 < - > ZH5 and CV1 < - > CV3) at multiple subpockets of protease active site. The challenge post-docking of the substrate peptides and mimics is to select realistic binding poses (i.e., P1′-P1-P3 substrate groups binding to S1′-S1-S3 subpockets) from a large number of docking poses (with random substrate groups at protease subpockets) which also need to show good interaction energies and anchor occupancies. For instance, substrate P4-P1 peptide VKRR (in Fig. 3D) is in the lowest energy docking pose but is unrealistic as P1 Arg is binding strongly at the S2 subpocket, P2 Arg fits in the S1 subpocket and P3 Lysine groups extending to the S1′ subpocket disobeying the realistic P4-P1-P1′ <−> S4-S1-S1′ binding principles. Also, the protease subpockets’ flexibility accommodating multiple substrate residues and the anchors preferring multiple moieties adds to more complications. We worked to resolve these issues by prioritizing docking poses similarity to the reference TGKR (crystal pose) in addition to best ASi and I.E values, to be the final substrate peptide binding poses.

The current target ZIKV NS3 protease has a wide druggable active site with distinct subpockets, that needs effective competitive inhibitors with moieties extending across sub-pockets, having higher anchor scores. In the current work, the PA model derived anchor-enhanced virtual screening, employing anchor scores was used for yielding ZIKV protease inhibitor candidates. So, the inhibitor candidate hits majorly contained high molecular weight FDA drugs (many of them showing high anchor occupancies); and some Maybridge, NCI and Natural product compounds with unique scaffolds and good anchor occupancies. This screening process using anchors is adaptable to refinements and can be modified to suit desired inhibitor discovery. Testing of the inhibitor candidates via *in vitro* virus production assays led to the discovery of FDA drugs Asunaprevir (EC_50_ = 4.7 µM) and Simeprevir (EC50 = 0.4 µM) as micromolar anti-ZIKV drugs. The inhibition of ZIKV NS2B/NS3 protease by these drugs was confirmed through *in vitro* enzyme-based assays at optimum assay conditions, Asunaprevir and Simeprevir showing dose-dependent inhibition of protease activities with IC_50_ values in micromolar range (6.0 µM and 2.6 µM respectively) equivalent with their efficacies observed in cell-based assays. These results highlight the immense repurposing potential of Asunaprevir and Simeprevir for ZIKV infections, which needs to be further investigated by *in vivo* studies. Also, the discovery of target-specific ZIKV protease inhibitors from ZIKV protease PA model based virtual screening validates the scope and potential of our drug discovery approach. Exploring the binding models of previr drugs reveals binding mechanisms of previr drugs interestingly similar to that of reference crystallized inhibitor 6T8. Moreover, interaction patterns of the previrs with ZIKV protease ‘His-Ser-Asp’ catalytic triad residues match to similar interactions in their original target HCV protease (observed in inhibitor bound HCV protease crystal structures: 4WF8 with Asunaprevir & 3KEE with Simeprevir). For example, a strong interaction of inhibitor –SO2-NH- functional groups with the catalytic Ser135 observed in ZIKV protease binding poses is the same as in HCV protease crystal bound structures, thus conforming the anchor-based inhibitor binding models to be credible.

One of the important challenges in current ZIKV protease drug discovery is achieving virus-specific protease inhibitors to avoid off-target interactions with human proteases^10^. We believe the PA model of ZIKV protease compared to that of human proteases, should reveal ZIKV specific anchors whose targeting would lead to virus-specific inhibitors with fewer side effects in humans. On the other hand, current observations from a comparative analysis of the flaviviral NS3 protease PA models (for ZIKV, DENV and WNV proteases) identified flaviviral protease core anchors which could be used as a guide map for highly desirable pan-flaviviral protease inhibitors^10^. Work in these directions is ongoing along with efforts to optimize anti-ZIKV protease inhibitors using anchors for improved potencies. In conclusion, our ZIKV NS3 protease PA model and anchors provides valuable understanding of viral protease mechanisms and facilitates the discovery of previr drugs for repurposing in anti-ZIKV treatments for accelerating therapeutic intervention of ZIKV infections.

## Materials and methods

### Multiple sequence alignment (MSA)

We collected protein sequences of NS2B cofactor and NS3 protease domains for mosquito-borne flaviviruses from UniProt: ZIKV (Q32ZE1), DENV (Q5UB51), WNV (P06935), JEV (O90417) and MVEV (P05769). MSA was performed using CLUSTAL Omega^46^ at default settings, which was then visualized by Jalview (http://www.jalview.org/). From the MSA we built evolutionary phylogenetic trees for both protease chains following the average distance method using the BLOSUM62 matrix.

### Protein-compound dataset preparation and docking

The crystal structures of the ZIKV NS3 protease were collected from the PDB database in both open (5T1V, 5TFN, 5TFO) and closed conformations (5GJ4, 5LC0, 5H6V, 5YOF, 5YOD, 5H4I, 5ZMQ, 5ZMS, 5ZOB)were aligned to reference 5GJ4 using CEAlign^38^. For the PA model, the active site of 5GJ4 was extracted by selecting protease residues within 12 Å of the TGKR ligand. For compound datasets, we collected Maybridge compounds (72057), Natural products (115683), NCI compounds with at least 1 ring per compound (50000 compounds) and FDA approved drug set (1384) from ZINCDB (https://zinc.docking.org/). For the PA model, we first docked a total of 2,37,740 compounds from all datasets into the extracted protease cavity using the GemDock^45^, at the standard docking settings with one pose per compound (10 poses for FDA drug set, one best pose selected). For binding mechanisms, we modeled the substrates P1-P4 peptides using the peptide builder module of the Avogadro molecular editor program (http://avogadro.cc/) followed by steepest descent and conjugate gradient minimizations until convergence. The substrate P1-P4 peptides were also docked at accurate docking settings with 10 poses per substrate peptides and the best pose selected based on similarity with reference TGKR.

### Building the PA model and calculating anchor scores

From the docking poses of the large compound datasets in the previous step, we selected top 3000 (~1.22%) compound poses with the best Interaction Energies (I.E) calculated by GemDock scoring function^45^. Their ‘protein residue – compound’ interaction profiles were analyzed and significant consensus E-H-V interactions (with cut-offs: Z-score > =1.65, I.E for E = H = < −2.5 kcal/mol and V = < −4 kcal/mol) were summarized as 3D anchors using analysis tool SiMMap^37^. The overlapping anchors in close vicinity were matched and merged according to the detailed criteria of the ‘anchor-matching rules’ elucidated in our previous work^27^, obtaining the final anchors (represented as mesh spheres). The total number of anchors occupied by a given compound is represented by the Anchor occupancy Score (AS_i_) calculated using the analysis tool SiMMap^37^. For Anchor scores (AS_i_) of substrate peptides, inhibitor candidates or other compounds bound to 5GJ4, a distance cutoff of 2.0 Å for anchor occupancy used, meanwhile for inhibitor crystal binding poses aligned to reference 5GJ4 anchors are calculated using a distance cutoff of 2.5 Å to accommodate residue chain conformational variability between crystal structures.

### MTT assay

In the MTT assays for cytotoxicities, cell cultures of Vero 76 (or U-87 MG) cells were plated on a 96-well plate and incubated overnight, followed by treatment with compounds or solvent (DMSO) only (n = 3–5). After 24 hours of incubation, 20 μl of MTT solution (5 mg/ml) was added to each well and incubated at 37 °C for 4 hours until crystal formation. The mixture was aspirated and Acid propan-2-ol (0.04 M HCl in isopropanol) was then added to each well to dissolve the dark blue crystals. We measured the absorbance as O.D. values at 570 nm (for all compounds, at all tested concentrations) by an ELISA reader (iMark™ Microplate Absorbance Reader, BioRad) from which % cell viabilities were calculated by comparing with the control. For inhibitors tested at multiple concentrations, the results were analysed by non-linear regression with a variable slope, to obtain dose-cell viability curves from which CC_50_ values were calculated (with GraphPad Prism V8.0).

### ZIKV fluorescence reporter assay

This assay employed a recombinant ZIKV with a fluorescent protein coding sequence integrated into its RNA genome and the virus replication efficiency was measured by the amount of emitted fluorescence using a fluorimeter. Here we used the MR766-Venus reporter virus (a gift from Dr. Matthew J Evans, Icahn School of Medicine at Mount Sinai), a ZIKV (MR-766 strain) tagged with Venus, a yellow constitutively fluorescent protein. Firstly, Vero 76 cells were cultured in a 96-well plate (2 × 10^4^ cells/well) for 16–24 hours. The cells were pre-treated with 5 µM of compounds and DMSO (control) for 2 hours, infected with MR766-Venus (MOI = 10) and incubated with the compounds (5 µM) for 24 hours (n = 5–6). The fluorescence intensities (directly corresponding to the viral count) were measured using a fluorimeter (SpectraMax M2, Molecular Devices) and graphs showing the % fluorescence intensities were plotted, for compound treatments and DMSO control.

### ZIKV production assay

In this virus production quantification assay, U-87 MG (human glioblastoma cell) cells were pre-treated with the inhibitors and DMSO (control) for 2 hours, infected with the ZIKV (PRVABC-59 strain, MOI = 1) and incubated for 24 hours in the presence of various inhibitor concentrations (0.0137 to 30 µM, 3x dilution) (n = 3). Then, the virus production in the culture supernatant was measured following the procedure of Focus forming assay described previously^47^. The results were plotted as graphs and the statistical significance was evaluated using one-way ANOVA for each treatment (inhibitor dose) compared to DMSO (control) by Dunnett’s multiple comparisons tests. From these results, we plotted dose-inhibition curves showing the effects of various inhibitor doses on virus titer (ffu/ml). The data were fitted using non-linear regression with a variable slope obtaining sigmoidal curves from which inhibitor EC_50_ values were calculated (with GraphPad Prism V8.0).

### ZIKV NS2B/NS3 protease expression, purification and enzyme inhibition assay

To express ZIKV NS2B/NS3 protease, the gene of ZIKV NS3 protease (residues 1–170) connected with the NS2B core region (residues 49–95) by a GGGGSGGGG linker was codon-optimized, synthesized (GenScript) and inserted into an expression plasmid vector pET28a pET28a using NcoI and XhoI restriction enzyme cutting sites. The recombinant plasmid was transfected into Rosetta2(DE3)pLysS (Novagen) cells and the clones positive for the plasmid were cultured overnight to an OD600 of 0.8 at 37 °C in LB medium and were then induced with 1 mM IPTG for 5 hours at 25 °C. The cells were harvested by centrifugation at 4 °C (6000 rpm, 10 min) followed by lysis by sonication in lysis buffer containing 1X PBS (137 mM NaCl, 10 mM Phosphate, 2.7 mM KCl, pH 7.4), 0.1% Triton X100 and 20 mM imidazole. The His-tagged ZIKV protease was purified by Ni Sepharose Fast Flow column (GE Healthcare) with a stepwise gradient of purification buffer (20 mM, 40 mM, 100 mM, 500 mM imidazole in 1X PBS). The purified ZIKV NS2B/NS3 protease was analyzed using sodium dodecyl sulfate polyacrylamide gel electrophoresis (10%), was aliquoted with approximately 95% purity and stored at −20 °C.

The protease activity tests were performed in a 96-well black flat-bottomed microtiter plates (Greiner Bio one, Germany) with a final volume of 100 µl. ZIKV NS2B/NS3 recombinant protease, at a final concentration of 5 nM, was pre-incubated for 15 min at 37 °C with either Asunaprevir and Simeprevir at different concentrations in the assay buffer (20 mM Tris pH7.5, 0.05% CHAPS, 1% Glycerol). The FRET substrate Benzoyl–Nle–KKR–AMC substrate was then added at a final concentration of 20 µM to the enzymatic reaction incubated for 30 min at 37 °C. The data for the same compound concentrations with the substrate and without enzyme were also measured as a control. The fluorescence signals (excitation/emission: 355 nm/460 nm) of the released AMC was measured using a fluorometer (VICTOR2, PerkinElmer). The results were plotted as dose-inhibition curves using non-linear regression with a variable slope to determine the IC50 values of inhibitor compounds (with GraphPad Prism V6.0). All assays were performed in duplicates in 96-well plates.

## Supplementary information

Supplementary Information .

## Data Availability

The PA model and anchor files generated during the current study are available in BioXGEM.GEMDOCK website, at http://gemdock.life.nctu.edu.tw/dock/download/ZIKV_NS3_protease_PA_model.zip
