# Supplementary material for: Zika Virus NS3 Protease Pharmacophore Anchor Model and Drug Discovery
Source: Sci Rep. 2020 Jun 2;10:8929. doi: 10.1038/s41598-020-65489-w (PMC7265434; doi:10.1038/s41598-020-65489-w)
Supplement: Supplementary file 1 — Supplementary Information . [file 41598_2020_65489_MOESM1_ESM.docx]

**Supplementary Information**

**Zika Virus NS3 Protease Pharmacophore Anchor Model and Drug Discovery**

**Nikhil Pathak^1,2^, Yi-Ping Kuo^3^, Teng-Yuan Chang^4^, Chin-Ting Huang^4^, Hui-Chen Hung^4^, John Tsu-An Hsu^4^, Guann-Yi Yu^3^, Jinn-Moon Yang^1,5,6,^***

^1^TIGP-Bioinformatics, Institute of Information Science, Academia Sinica, Taipei, Taiwan

^2^Institute of Bioinformatics and Structural Biology, National Tsing Hua University, Hsinchu, Taiwan

^3^National Institute of Infectious Diseases and Vaccinology, National Health Research Institutes, Zhunan, Taiwan

^4^Institute of Biotechnology and Pharmaceutical Research, National Health Research Institutes, Zhunan, Taiwan

^5^Institute of Bioinformatics and Systems Biology, National Chiao Tung University, Hsinchu, Taiwan

^6^Department of Biological Science and Technology, National Chiao Tung University, Hsinchu, Taiwan

*Correspondence should be addressed to J.M.Y. (email: [moon@faculty.nctu.edu.tw](mailto:moon@faculty.nctu.edu.tw))

**Supplementary Table S1. ZIKV NS3 protease inhibitors and substrate peptides - Interaction energies and anchor occupancies.** The summary of binding features like interaction energies, anchor scores and compound moieties occupying ZIKV protease PA model anchors for binding poses of (A) Inhibitors (B) Substrate peptides. ^a^interaction energy scoring function not applicable.

| **Compound** | **I.E (kcal/mol)** | **AS_i_** | **Compound moieties at anchors** | | | | | | | | | | | |
| --- | --- | --- | --- | --- | --- | --- | --- | --- | --- | --- | --- | --- | --- | --- |
|  |  |  | **CEH1** | **CH3** | **CH7** | **CV1** | **CV3** | **ZEH4** | **ZEV2** | **ZH2** | **ZH5** | **ZH6** | **ZV4** | **ZV5** |
| 1. **Inhibitors from PDB** | | | | | | | | | | | | | | |
| 6T8 (5LC0) | -^a^ | 9 | 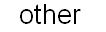 | 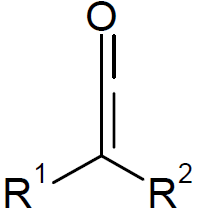 | - | 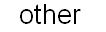 | 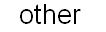 | 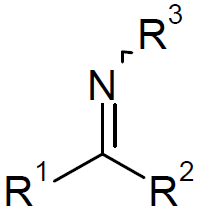 | 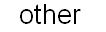 | - | 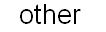 | - | 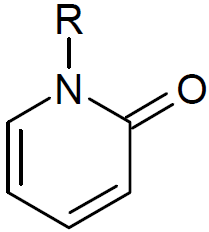 | 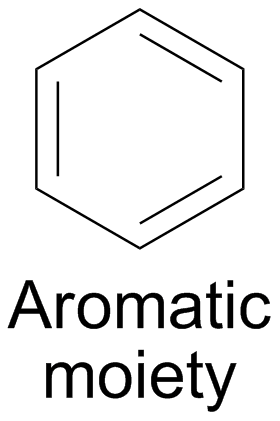 |
| 7HS (5H6V) | -104.9 | 10 | 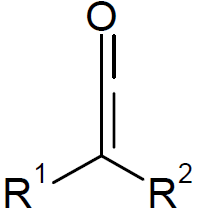 | 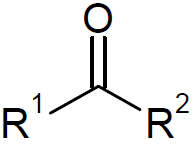 | 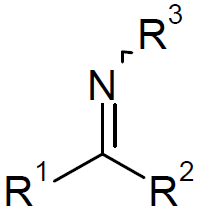 | 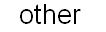 | 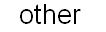 | 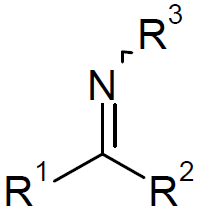 | 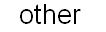 | - | 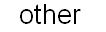 | - | 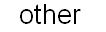 | 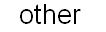 |
| 7HS (5YOF) | -72.7 | 10 | 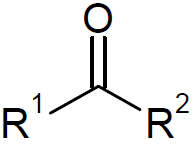 | 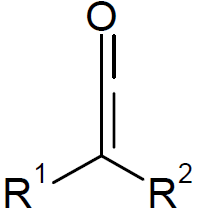 | 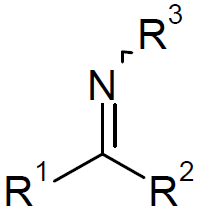 | 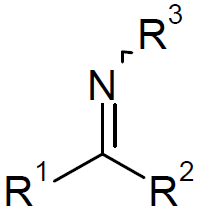 | 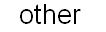 | 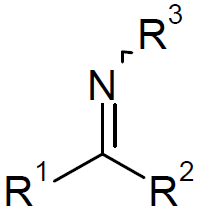 | 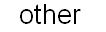 | - | 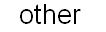 | - | 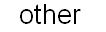 | 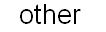 |
| BEZ (5YOD) | -26 | 4 | 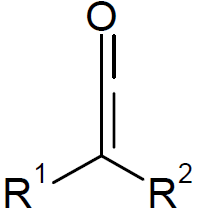 | 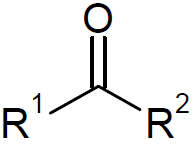 | - | 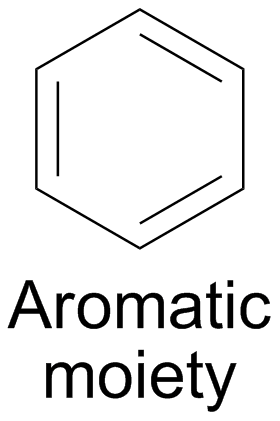 | - | - | 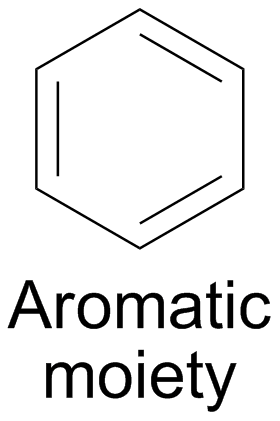 | - | - | - | - | - |
| 7HQ (5H4I) | -38.4 | 3 | - | - | - | 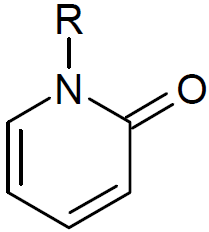 | - | - | 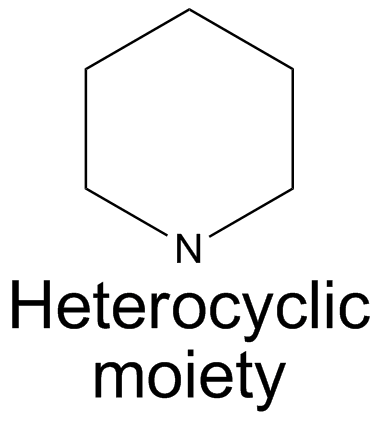 | 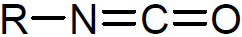 | - | - | - | - |
| C1 (5ZMQ) | -113.6 | 10 | 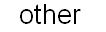 | 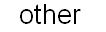 | 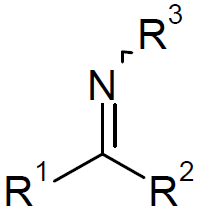 | 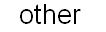 | 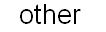 | 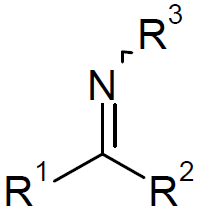 | 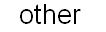 | - | 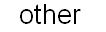 | - | 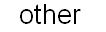 | 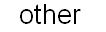 |
| C2 (5ZMS) | -123.6 | 8 | - | 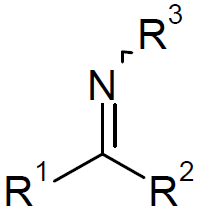 | - | 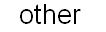 | 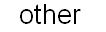 | - | 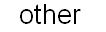 | 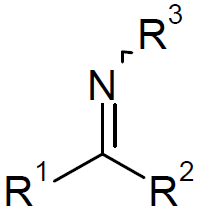 | 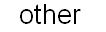 | - | 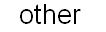 | 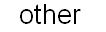 |
| C3 (5ZOB) | -145.1 | 5 | - | - | - | 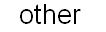 | 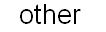 | - | 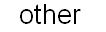 | - | - | - | 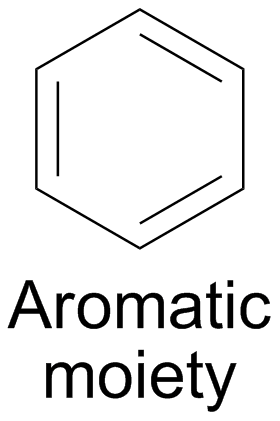 | 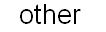 |
| 1. **Substrate peptides** | | | | | | | | | | | | | | |
| TGKR (5GJ4) | -118.6 | 5 | - | - | - | 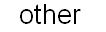 | 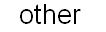 | - | 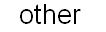 | - | - | - | 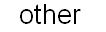 | 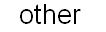 |
| VTRR | -160.6 | 8 | 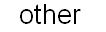 | 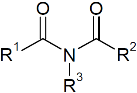 | 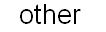 | 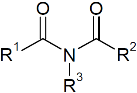 | 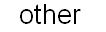 | - | 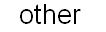 | 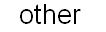 | - | - | - | 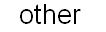 |
| SGKR | -120.6 | 7 | 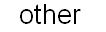 | 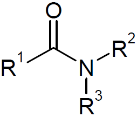 | 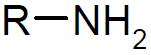 | 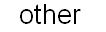 | 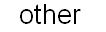 | - | 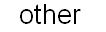 | - | 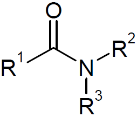 | - | - | - |
| AGKR | -143.2 | 10 | 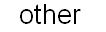 | 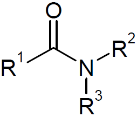 | 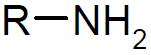 | 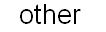 | 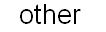 | 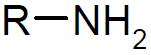 | 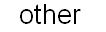 | - | 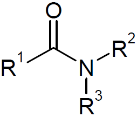 | - | 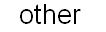 | 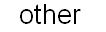 |
| VKRR | -149.2 | 10 | 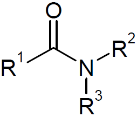 | 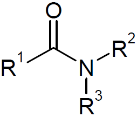 | 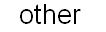 | 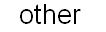 | 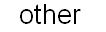 | 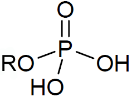 | 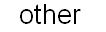 | 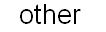 | 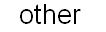 | - | 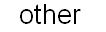 | - |
| Bez- VKKR -H | -157.6 | 9 | - | 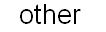 |  |  |  |  |  |  | - |  | - |  |

**Supplementary Table S2. ZIKV NS3 protease inhibitor candidate compounds - Interaction energies and anchor occupancies.** For the inhibitor candidate binding poses the interaction energies, anchor scores and compound moieties occupying the ZIKV protease PA model anchors are summarized.

| **Compound** | **I.E (kcal/mol)** | **AS_i_** | **Compound moieties at anchors** | | | | | | | | | | | |
| --- | --- | --- | --- | --- | --- | --- | --- | --- | --- | --- | --- | --- | --- | --- |
|  |  |  | **CEH1** | **CH3** | **CH7** | **CV1** | **CV3** | **ZEH4** | **ZEV2** | **ZH2** | **ZH5** | **ZH6** | **ZV4** | **ZV5** |
| Artesunate | -102.3 | 4 | - | - |  |  |  | - |  | - | - | - | - | - |
| Dasatinib | -117.9 | 7 | - |  |  |  |  |  |  | - |  | - | - | - |
| Losartan | -117.1 | 8 | - |  |  |  |  |  |  |  | - | - | - |  |
| Ritonavir | -186.2 | 8 | - |  |  |  |  | - |  |  | - | - |  |  |
| Saquinavir | -153.4 | 8 |  | - |  |  |  |  |  | - | - | - |  |  |
| Amprenavir | -148.4 | 8 | - |  |  |  |  | - |  |  |  | - | - |  |
| Nelfinavir | -144.1 | 9 |  |  |  |  |  |  |  | - | - | - |  |  |
| Boceprevir | -141.9 | 9 |  |  |  |  |  | - |  | - |  | - |  |  |
| Asunaprevir | -135.8 | 8 |  |  | - |  |  | - |  | - |  | - |  |  |
| Simeprevir | -110.3 | 7 |  |  | - |  |  | - |  | - | - | - |  |  |
| VMSTK 046507 | -118.3 | 8 | - |  |  |  |  | - |  | - |  | - |  |  |
| NAT18-355744 | -125.8 | 5 | - |  |  |  |  |  | - | - | - | - | - | - |
| NSC146495 | -142.1 | 7 | - |  | - |  |  | - |  |  |  |  | - | - |

**Supplementary Figure S1. Sequence-structure analysis of ZIKV NS3 protease with other flaviviral proteases.** (A) MSA and phylogenetic trees comparing ZIKV NS2B cofactor and NS3 protease sequences to that of other flaviviruses (residues colored blue based on % identity, motifs underlined orange, catalytic residues highlighted by *). (B) Structures of ZIKV NS2B/NS3 proteases in closed and open forms.

**Supplementary Figure S2. PA models of ZIKV NS3 protease vs other flaviviruses.** (A) ZIKV protease PA model alignment with that of DENV, WNV and JEV proteases followed by anchor matching revealing ZIKV core anchors (CEH1, CH3, CH7, CV1 and CV3, magenta dotted circles and text) and specific anchors. (B) Anchor matching among ZIKV, DENV and WNV NS3 protease PA models with core anchors (magenta dotted arrows) and specific anchors (brown dotted arrows). (C) Anchor matching summary.

**Supplementary Figure S3. Comparative analysis of core and specific anchors in NS3 proteases of ZIKV, DENV and WNV.** (A) Five flaviviral core anchors with anchor features like interaction types, aligned anchor residues and consensus moiety preferences, colored ZIKV (salmon), DENV (yellow) & WNV (cyan). (B) Specific anchors of ZIKV, DENV and WNV proteases with anchor features. Anchors are shown as mesh spheres (colored acc. to E-H-V types); anchor residues shown (matching - sticks, non-matching – lines).

**Supplementary Figure S4. Anchor residue conservation analysis.** (A) % Residues with conservation scores for all anchor residues (core and specific), other binding site residues and other residues. (B) % Residues with conservation scores for core and specific anchors.

**Supplementary Figure S5. Binding poses of inhibitor candidates with the ZIKV protease PA model.** The ZIKV protease active sites (5GJ4) with 14 inhibitor candidate poses are shown, containing 10 FDA drugs (blue outline), one Maybridge compound (orange outline), one natural product (green outline) and two NCI compounds (purple outline).
